# For the State Cancer Laboratory

**Published:** 1911-02

**Authors:** 


### For the State Cancer Laboratory

SENATOR LOOMIS of Buffalo, has introduced into the Legislature a bill appropriating $65,000 for the building of a cancer hospital of 25 bed capacity on the property adjoining the New York State Cancer Laboratory opposite the Buffalo General Hospital on High street.

The bill was introduced on January 19, 1910, and its introduction was followed by a statement by the Director of the Laboratory, Dr. H. R. Gaylord, which as reported in the daily press, was as follows:

"Yes, a cure has been effected," said Dr. Gaylord, "and the work of the laboratory has progressed to the point where it is absolutely necessary that we should have a research hospital in connection with the institution if we are to keep abreast of what is being done elsewhere. Our experiments with vaccination have brought results that justify us in applying the treatment to human beings in a hospital of this kind. In fact we have treated a number successfully in addition to the case of the boy cured.

"At the Cancer Congress in Paris last October there were reported six cures, two from Copenhagen, two from Rome, one from Brussels and the one from Buffalo. Several of these went there, each believing he was the first and not knowing what the others had accomplished."

The case referred to is reported to be that of a seventeen-year old boy who was afflicted with "cancer" of the neck just below the jaw.

The exact nature of the growth is not stated, the newspapers merely being content with stating it to be a "cancer." Whether it be cancer or not makes little difference. There is no question raised as to its malignancy and that fact alone is deemed of sufficient importance to warrant the state expending money for further investigation.

A news dispatch from Albany states that the bill provides that the state shall appropriate $65,000 for putting up a building for hospital research purposes on the site adjoining the laboratory. Five citizens of Buffalo have guaranteed to raise $20,000 for the land and the state appropriation is made on the expressed condition that title to this site, which is 150 x 179 feet, together with title to the Gratwick Laboratory property, building and equipment shall pass to New York State. The entire institution would be called the New York State Institute for the Study of Malignant Diseases. The laboratory was built by Mrs. William H. Gratwick of Buffalo and title to it is nominally vested in the University of Buffalo. The purposes of the new institute are set forth in the bill as follows:

"This institute shall conduct investigation into the causes, nature, mortality rate, treatment, prevention and cure of cancer and allied diseases, and may receive in the hospital for study, experimental or other treatment, cases of cancer and allied diseases free of charge. It shall publish from time to time the results of this investigation for the benefit of humanity and shall from time to time collect it and publish in the form of a scientific report for distribution."

A further provision is for the appointment of a board of trustees, and those named for the first board are as follows:

State Commissioner of Health, ex-officio; Dr. Roswell Park of Buffalo, John G. Milburn of New York, Frederick C. Stevens of Attica, Dr. Charles Cary of Buffalo, Dr. Charles S. Fairchild of New York, and William H. Gratwick of Buffalo.

---

INCREASING numbers of soiled bills are being returned to Washington for redemption. Mr. A. Cressy Morrison, of Chicago (*Morning Telegraph*) still carries on with enthusiasm a campaign against "filthy lucre;" filthy in the literal sense of the word when represented by the bills commonly used in present-day transaction of business.

The results of a thorough analysis of twenty-four of the worst bills turned in are startling. There were germs of grippe, tuberculosis, diphtheria and other contagious diseases, says the *National Magazine*. Mr. Morrison claims that the examination made for typhoid and cholera germs in drinking water should be applied to bills. When Mr. Hilditch, of Yale, examined twenty-four bills he discovered an average of 142,000 bacteria of various dangerous maladies, which is a matter of great concern when it is considered that this money had passed through the hands of thousands of men, women and children all over the country. It has been proved scientifically that paper money is a means of transmission of disease. Germs of tuberculosis and other contagious disorders may live for several days in bills. From a sanitary standpoint the Federal government ought to remedy this disgraceful condition. In fact, the bills become so contaminated that no self-respecting person would accept such an accumulation of filth in any other form than money in a public or private transaction.

---

THOSE rotten eggs, reference to which was made in the January issue of the JOURNAL, have been perplexing the authorities ever since, or at least until very recently. Dr. Fronczak, the health commissioner, has maintained a guard over the cold storage building where the eggs were kept since they were condemned until lately, but this is expensive, so the corporation counsel was appealed to for a remedy. He says the health commissioner has no authority under the charter or ordinances to destroy eggs or other products condemned as unfit for food purposes. Recently a quantity of eggs in cold storage was condemned by Dr. William H. Heath of the health department as unfit for food. These eggs were in cold storage for some time.

Corporation Counsel Hammond says the only procedure he can suggest to the health commissioner is to hold a hearing, at which the owner of the eggs and the owner of the warehouse may present their side of the case. He suggested that the charter should be amended to give the health commissioner more power in such cases. A hearing was determined upon at which the parties at interest were cited. Meanwhile the owners of the eggs consented that the health commissioner might turn them over to a tannery. So much for an old-fashioned charter.

